# Spatial and temporal Antarctic Ice Sheet mass trends, glacio‐isostatic adjustment, and surface processes from a joint inversion of satellite altimeter, gravity, and GPS data

**DOI:** 10.1002/2015JF003550

**Published:** 2016-02-03

**Authors:** Alba Martín‐Español, Andrew Zammit‐Mangion, Peter J. Clarke, Thomas Flament, Veit Helm, Matt A. King, Scott B. Luthcke, Elizabeth Petrie, Frederique Rémy, Nana Schön, Bert Wouters, Jonathan L. Bamber

**Affiliations:** ^1^ School of Geographical SciencesUniversity of BristolBristolUK; ^2^ Centre for Environmental Informatics, National Institute for Applied Statistics Research AustraliaUniversity of WollongongWollongongNew South WalesAustralia; ^3^ School of Civil Engineering and GeosciencesNewcastle UniversityNewcastle upon TyneUK; ^4^ LEGOSToulouseFrance; ^5^ Alfred Wegener InstituteBremerhavenGermany; ^6^ School Land and FoodUniversity of TasmaniaHobartTasmaniaAustralia; ^7^ NASA Goddard Space Flight CenterGreenbeltMarylandUSA; ^8^ School of Geographical and Earth SciencesUniversity of GlasgowGlasgowUK

**Keywords:** Antarctica, Mass balance, SMB, ice dynamics, GIA

## Abstract

We present spatiotemporal mass balance trends for the Antarctic Ice Sheet from a statistical inversion of satellite altimetry, gravimetry, and elastic‐corrected GPS data for the period 2003–2013. Our method simultaneously determines annual trends in ice dynamics, surface mass balance anomalies, and a time‐invariant solution for glacio‐isostatic adjustment while remaining largely independent of forward models. We establish that over the period 2003–2013, Antarctica has been losing mass at a rate of −84 ± 22 Gt yr^−1^, with a sustained negative mean trend of dynamic imbalance of −111 ± 13 Gt yr^−1^. West Antarctica is the largest contributor with −112 ± 10 Gt yr^−1^, mainly triggered by high thinning rates of glaciers draining into the Amundsen Sea Embayment. The Antarctic Peninsula has experienced a dramatic increase in mass loss in the last decade, with a mean rate of −28 ± 7 Gt yr^−1^ and significantly higher values for the most recent years following the destabilization of the Southern Antarctic Peninsula around 2010. The total mass loss is partly compensated by a significant mass gain of 56 ± 18 Gt yr^−1^ in East Antarctica due to a positive trend of surface mass balance anomalies.

## Key Points

- Simultaneous estimate of ice dynamics, SMB and GIA for the Antarctic Ice Sheet,
- The total mass loss of the AIS is −84 ± 24 Gt yr^−1^ over 2003–2013
- West Antarctica is the largest contributor triggered by high thinning rates of glaciers in the ASE

## 1 Introduction

Antarctica has been losing mass over the last two decades, probably at an accelerating rate [*Williams et al.*, 2014; *Harig and Simons*, 2015]. Quantifying the losses, their glaciological origin, and their spatiotemporal characteristics remains an important goal for closing the recent sea level budget and as an input into, and constraint for, numerical models that attempt to improve process understanding and predict future behavior of the ice sheet. Various approaches exist for estimating the Antarctic Ice Sheet (AIS) mass balance, each of which has its own strengths and weaknesses. Three approaches are most commonly used: (i) estimating volume changes using satellite altimetry [e.g., *Zwally et al.*, 2005] and converting these to a mass change, (ii) the input‐output method (IOM) [e.g., *Rignot et al.*, 2008] that compares ice discharge across (usually) the grounding line with snow accumulation over the upstream catchment area, and (iii) conversion of gravity anomalies from the Gravity Recovery and Climate Experiment (GRACE) satellites into mass trends. All of these methods rely on forward models to solve for an unmeasured or unknown process that influences the observation. In the case of altimetry, the conversion from a volume change to a mass change usually uses a regional climate model (RCM) to solve for firn compaction and surface density. In the case of the IOM, a RCM is normally used to determine the snow accumulation term, and in the case of gravimetry, forward models are often used to determine the solid‐Earth signal due to glacio‐isostatic adjustment (GIA). These forward models have hard‐to‐quantify biases that likely adversely affect the solution [cf., *Schoen et al.*, 2015; *Zammit‐Mangion et al.*, 2015a]. As a consequence, there are inconsistencies in the spatiotemporal mass trends not only between these approaches but also between the different estimates obtained using the same approach. Unfortunately, simple arithmetic means of the combined estimates [*Shepherd et al.*, 2012] mask the large differences between individual estimates and do not account for common sources of error. Combining estimates, in a statistically rigorous way, is challenging because satellite gravimetry, altimetry, and the IOM have markedly different spatial and temporal resolutions and error covariances.

In this paper, we take an approach that accounts for the different spatiotemporal properties of the various data sets and physical processes via a Bayesian hierarchical framework. This enables us to solve simultaneously for the relevant processes that are being observed and, from these, to derive the mass trends. Specifically, we solve for the following latent physical processes: (1) mass trends due to changes in ice dynamics (hereafter referred to as ice dynamics), (2) surface mass balance (SMB) anomalies from an equilibrium state, (3) firn compaction and viscous‐elastic rebound of the solid Earth which can be approximated as the sum of two separate components: (4) the long‐wavelength viscous response referred as GIA, and (5) the instantaneous elastic response. Four of these processes are solved for in the simultaneous inversion, while elastic rebound is determined iteratively as detailed in section 2.3 using a forward modeling approach. GIA is time invariant, but all other processes vary in both space and time. Here we focus on the drainage basin‐scale, annually resolved mass trends for the period 2003–2013 inclusive. We note, however, that there is valuable and important information contained in all five fields, which can aid interpretation of the trends and process understanding. In this study, we utilize satellite altimeter data from Envisat, ICESat, and CryoSat‐2, gravimetry data from the GRACE mission, and in situ GPS measurements of vertical bedrock motion.

## 2 Data Sets

Multiple types of satellite and in situ measurements are used in this study to determine the mass trends of the AIS. Additionally, we employ auxiliary data from numerical models to extract the characteristic length scales of the geophysical processes driving the elevation changes. A summary of the data sets included in this study is shown in Table 1.

**Table 1:** Summary of Data Sets Used in This Study

Observation	Processes	Time Period	Reference
ICESat	SMB, ICE, FIRN, and GIA	2003–2009	NSIDC
Envisat	SMB, ICE, FIRN, and GIA	2003–2010	*Flament and Rémy* [2012]
CryoSat‐2	SMB, ICE, FIRN, and GIA	2010–2013	*Helm et al.* [2014], *Wouters et al.* [2015]
GRACE	SMB, ICE, and GIA	2003–2013	*Luthcke et al.* [2013]
GPS	GIA	2003–2009	*Groh et al.* [2012], E. Petrie (Glasgow Univ.)
INSAR	Horizontal ice velocities	–	*Rignot et al.* [2011]
RACMO	SMB	1979–2009	*Lenaerts et al.* [2012]
Firn model	Firn	1979–2009	*Ligtenberg et al.* [2011]

### 2.1 Altimetry

We use three altimetry data sets to obtain time series of elevation change for the period 2003–2013. Along‐track elevation rate measurements from ICESat, data release 33, provide high‐resolution altimetry observations for the period February 2003 to October 2009. Data were corrected for the range determination from Transmit‐Pulse Reference‐Point Selection (Centroid versus Gaussian) [*Borsa et al.*, 2014] available from the National Snow and Ice Data Center (NSIDC) and for the intercampaign bias presented in *Hofton et al.* [2013]. Yearly trends of elevation changes and their associated errors were calculated, from a 3 year moving window centered on the year under consideration, using a “plane regression” approach [*Howat et al.*, 2008; *Moholdt et al.*, 2010] in which both spatial and temporal slopes were simultaneously estimated over areas spanning 700 m long and a few hundred meters wide. Regression was only performed if a minimum of 10 points from four different tracks spanning at least a year was available. Mean annual trend estimation was performed in two steps: first a regression was carried out to detect and remove outliers falling outside the 2 standard deviation (2*σ*) confidence interval and then the annual trend and its standard deviation was determined. To avoid noisy spatial patterns, only elevation changes with an associated error smaller than 0.40 m yr^−1^ were considered. These annual trends were averaged over a regular Cartesian 20 km grid, and the standard deviations of the trends within each grid cell were taken as an estimate of the measurement error.

Envisat radar altimetry data were available for the period January 2003 to November 2010. These data provide better temporal and spatial coverage than ICESat for much of the AIS. However, their spatial resolution is poorer than ICESat and they are, therefore, complementary. Elevation trends were obtained at points every 1 km along track, by binning all the echoes within a 500 m radius and then fitting a 10‐parameter least squares model to the entire Envisat interval in order to correct for the across‐track topography and changes in snowpack properties [*Flament and Rémy*, 2012]. For consistency with ICESat, annual time series were produced using a 3 year moving window and resampled onto a 20 km grid, approximating the observation error as the standard deviation of the data points within each grid cell.

CryoSat‐2 provides high spatial resolution radar altimetry data (July 2010 to December 2013) up to a latitude of 88°S. In addition, it performs more reliably in coastal areas where Envisat and previous radar altimeters struggle. Elevation rates were derived from Level 1B data processed as described in *Helm et al.*[2014], using an in‐house Level 2 processing chain that includes a waveform filter and a threshold first maximum retracker, specifically developed to reduce the effect of subsurface scattering of the radar echo. To estimate the surface elevation trends, we used a pseudorepeat track method, where rates are estimated at every CryoSat‐2 echo location by fitting a model of surface topography and elevation change to all neighboring echoes within a 1 km radius [*Wouters et al.*, 2015]. As for the other altimetry data sets, the elevation rates at the individual locations were then averaged in 20 × 20 km grid cells, removing outliers using a 2*σ* filter. The errors of the elevation rates were estimated from the standard deviation within the grid cell. Both trends and accelerations were estimated for the 3.5 year period. We integrated multiyear trends in our framework, assuming that observation errors are uncorrelated, by upscaling the error on the unknown as $\sigma \sqrt{n}$, where *n* is the number of times we are using the observation and *σ* the observation error. We aim at determining areas where the acceleration term (if present) is due to ice dynamics and not SMB. To identify these regions, we estimated the average rate of elevation change (d*h*/d*t*) due to surface processes from a regional climate model, Regional Atmospheric Climate Model version 2.3 (RACMO2.3) [*van Wessem et al.*, 2014], for the period 1979–2013 and compared them against those recorded by CryoSat‐2. Annual rates of elevation change were derived by including the acceleration terms in those regions where the rate of height change was larger than 2*σ* of the SMB‐driven elevation changes from RACMO and where ${\bar{R}}_{\text{acc}}^{2}$ ≥ ${\bar{R}}_{\text{linear}}^{2}$, where ${\bar{R}}_{\text{acc}}^{2}$ and ${\bar{R}}_{\text{linear}}^{2}$ are the adjusted coefficient of determination of the quadratic and linear fits, respectively. If these criteria were not met, only the linear 3 year trend was used. To cope with the small SMB variability in the interior of Antarctica, where the elevation changes are not significantly different from zero, we used an ice surface velocity threshold of 25 m yr^−1^ as a lower bound for permitting an acceleration term.

### 2.2 Gravimetry

In this study we use the latest release of the NASA Goddard Space Flight Center global mass concentration (mascon) solution, RL2 v15 (unpublished), for the period January 2003 to December 2013. This solution is based on *Luthcke et al.* [2013] with a number of improvements, including new release 2 GRACE Level‐1 data, improved altitude and accelerometer data anomaly handling and editing, improved K band range rate (KBRR) observation editing, improved accelerometer and KBRR arc parameter strategy, and improved forward models. The present mascon solution is a multi‐iterated convergence which helps to recover more signal and reduces noise. Improvements in the forward models used compared to those in the *Luthcke et al.* [2013] study include MOG2D ocean model, Paulson‐ICE5G GIA model (GIA was forward modeled and then restored), Goddard Ocean Tide 4.7 tides modeled to degree and order 90, International Earth Rotation Service (IERS) 2010 pole and solid earth tides, and DE421 planetary ephemeris. The global set of 1 × 1 arc‐degree equal area mascons have been directly estimated monthly from the reduction of the GRACE KBRR data applying separate spatial anisotropic constraints to land, ocean, and land ice regions as in *Luthcke et al.* [2013]. However, RL2 v15 applies a single region constraint for Antarctica land ice and therefore does not use the 2000 m high/low‐constraint regions as in *Luthcke et al.*[2013]. The RL2 v15 mascon solution is corrected for the poorly resolved degree 2 and the unobserved degree 1 contribution [*Luthcke et al.*, 2013]. To remove the correlation among the observations, we averaged the signal on a 300 km polar grid [*Zammit‐Mangion et al.*, 2015a]. Subannual variability was identified and removed using the ensemble empirical mode decomposition adaptive filtering approach described in *Loomis and Luthcke* [2014] and *Luthcke et al.* [2013]. Linear trends were then fitted to each year to obtain annual mass trends for each mascon. Error estimates were determined from the regression errors of the linear fit. We assume that GRACE does not observe SMB or other ice mass changes over the floating ice shelves as they are in hydrostatic equilibrium. Hence, all observed mass changes inferred over the ice shelves were assumed to be caused by GIA.

### 2.3 GPS

GPS analysis was performed as in *Thomas et al.* [2011] with a number of updates and exceptions. For each day we processed a global network of around 80 GPS sites, located on the rock. Sites were selected to achieve the broadest global distribution for producing precise satellite orbits and clocks. These orbits and clocks were then used in precise point positioning mode to generate coordinate time series for Antarctic sites of interest. The processing software used was GIPSY/OASIS v6.2; Earth radiation and second‐order ionospheric error models (shell height 600 km) were applied; the time period processed was 1995.0–2013.7; satellite and receiver antenna phase centers were calibrated according to the igs08_1748.atx values; Earth tides were modeled as described in the IERS2010 conventions [*Petit and Luzum*, 2010]; station coordinates were transformed from the daily nonfiducial reference frame into ITRF2008 using Helmert parameters defined by a set of 40 sites selected each day for maximum hemispheric balance; ambiguities were fixed to integers using the GIPSY routine *edtpnt2*, and subdaily atmospheric loading models were not applied, but atmospheric loading was later removed from the daily solutions [*Santamaría‐Gómez and Mémin*, 2015].

We chose to solve for GIA over the 2003–2009 period, as this represents the most complete epoch in terms of coincident data sets (ICESat, ENVISAT, GPS, and GRACE data). The GIA signal is assumed to be temporally invariant on the decadal time scale of this study. We calculated a set of annual vertical bedrock velocities at each GPS site where at least 3 years of good quality continuous data with no significant gaps were available. This was done using an algorithm developed to avoid spurious trends from noisy time series. First, seasonal signals were removed by subtracting the monthly means from the GPS coordinate data prior to finding the trend. Then, annual trends were estimated, fitting a piecewise linear function to the data at each site to avoid discontinuities at the year boundaries. For further details of the algorithm, see https://andrewzm.wordpress.com/2013/12/12/estimating-yearly-trends-with-an-em-algorithm-in-r/.

Non‐ideal sites, consisting of campaign rather than continuous GPS data or with large portions of data missing, are unsuitable for annual trend estimation. At these sites a single linear trend was estimated. Such sites were only included if they contained some data during 2003–2009, but due to the temporal and spatial sparsity of Antarctic GPS stations, available data from outside the period were also used. To achieve more realistic trends and confidence intervals at these sites, median noise level estimates found using the Computer Aided Transcription System software [*Williams*, 2008] from Antarctic sites with over 2000 daily solutions were used for error propagation. These suboptimal time series were further assessed by manually removing random portions of the time series and calculating the variability in the estimated trends using *tsview* (GGMatlab tools) [*Herring*, 2003]. If the trend changed by an amount larger than the propagated confidence interval for the site, the latter was increased to the maximum difference in the observed trend. In a few cases, where removing an individual campaign led to large changes in trend, the adopted trend was changed to one better reflecting the majority of the data. As such checking is not possible for sites with only two campaigns, the confidence interval at these sites was inflated so that it reflected the possibility of systematic campaign‐specific error affecting the rate estimation. Finally, published trends from the three sites in *Groh et al.* [2012] in the Amundsen Sea Embayment (ASE) sector were also included, with the confidence limits from the paper treated as for the other two‐campaign sites. In this particular region which is affected by a strong elastic signal, we took a conservative approach and considered 100% of the original error as the uncertainty in the elastic correction (as explained below) which was used as an upscaling factor for the GPS errors. The magnitudes of the uncertainties of the GPS rates in the ASE sector are on the order of 3–4 mm yr^−1^ (the original uncertainty stated in *Groh et al.* [2012] was 1.7 mm yr^−1^). Each linear trend was then effectively considered as a set of annual trends with a constant value over the period 2003–2009.

Once the annual vertical trends had been estimated, they were corrected for elastic rebound due to present‐day ice load changes. Elastic deformations are caused by instantaneous or short period processes, such as snowfall or ice discharge events, while a viscoelastic deformation occurs over longer time scales (from decades to thousands of years), in which the mantle is redistributed [*Klemann et al.*, 2014]. To correct for the instantaneous elastic response, we used an iterative approach. First, we ran the Bayesian framework explained in section 3 excluding the GPS station data to obtain an initial estimate of the load changes caused by SMB anomalies and ice dynamics. The effects of these loading changes on the vertical motion of the Earth's crust were calculated using the Regional Elastic Rebound Calculator (REAR) [*Melini et al.*, 2015]. In order to compute the surface rates of displacement associated to a unit rate of mass variation (Green functions), REAR uses a set of load deformation coefficients from the seismological model *STW105* [*Kustowski et al.*, 2007] up to maximum value of 30,000 harmonic degrees. Vertical displacements were calculated following the methods described in *Melini et al.* [2015] and used to correct the GPS trends. Then, the framework was run again incorporating the elastic‐corrected GPS trends. This process was iterated twice. It is well known that in East Antarctica, a whole year's anomaly can be caused by only a few snowfall events [*Boening et al.*, 2012]. If the GPS time series are not corrected for the elastic response due to these anomalies, the underlying GIA signal can be estimated incorrectly.

### 2.4 Additional Data Sets

We used auxiliary data sets (both observational and extracted from geophysical models) to help constrain our solution by extracting information on the spatial and temporal smoothness of the different processes driving AIS mass changes. The Regional Atmospheric Climate Model (RACMO2.1/ANT27) [*Lenaerts et al.*, 2012] was used to analyze the spatiotemporal properties of SMB anomalies over the 2003–2009 period (with respect to the 1979–2002 mean). A firn densification model [*Ligtenberg et al.*, 2011] and the regional climate model were used to undertake a correlation analysis between changes in the firn thickness and precipitation. Ice surface velocities were obtained from a satellite interferometry data set [*Rignot et al.*, 2011] and theoretical balance velocities [*Bamber et al.*, 2000] and used to constrain the maximum change in height due to ice dynamics under the assumption that higher dynamical mass losses will likely occur in areas with large horizontal velocities [*Hurkmans et al.*, 2012].

## 3 Bayesian Framework

The Bayesian hierarchical model (BHM) that is used in this study is a new approach to the problem of separating the different components comprising the mass balance of an ice sheet although the generic problem of source separation has been tackled using similar approaches in a wide range of other fields. This methodology has been previously presented and tested in four preceding papers: (1) *Zammit‐Mangion et al.* [2014]; (2) *Schoen et al.* [2015]; (3) *Zammit‐Mangion et al.* [2015a]; and (4) *Zammit‐Mangion et al.* [2015b]. The first introduces the mathematical details, concepts, and principles of the time‐invariant BHM. The second applies the time‐invariant BHM to estimate the mass balance of West Antarctica, compares the obtained estimates with other results, and presents sensitivity analyses to length‐scale choices. The third includes the temporal variability and constitutes the current version of the BHM used in this work. A thorough explanation of the spatiotemporal modeling tools, and how numerical models have been used to obtain the spectral characteristics, both in space and time, of the different processes is presented in that study. The fourth provides the conceptual principles underlying our approach to source separation and the structure of the BHM in a nonmathematical style, applying the concepts to a toy model example illustrating how spatial and/or temporal smoothness information can be used to aid source separation. We do not, therefore, attempt to replicate that material here but aim to summarize the development of the methodology. The focus of this paper is on the results obtained using our approach rather than the approach itself, which has been comprehensively described elsewhere.

In the present study, we use the spatiotemporal BHM to estimate the mass trends of the AIS for 2003–2013 but with some subtle improvements with respect to the approach detailed in *Zammit‐Mangion et al.* [2015b]. First, the characteristic GIA length‐scale is allowed to vary spatially: in the previous studies [*Schoen et al.*, 2015; *Zammit‐Mangion et al.*, 2015b] this was fixed at 1800 km following a spectral analysis of a forward model solution for GIA [*Ivins et al.*, 2013]. This fixed value is, however, less representative of the rheology of West Antarctica and, in particular, the Antarctic Peninsula where shorter wavelength variations have been predicted due to the weaker mantle rheology of the sector [*Ivins et al.*, 2011; *Nield et al.*, 2014]. Here we permit the length scale to vary spatially, growing from an empirical value of 500 km at the AP where the upper mantle viscosities are lower [*Nield et al.*, 2012] to a maximum of 1700 km across East Antarctic Ice Sheet (EAIS). Second, because of the longer time period covered here, we approximate the dynamic imbalance as a quadratic accelerating/decelerating temporal trend, as we found that a linear temporal trend poorly represents the changes in regions such as the Amundsen Sea Embayment and the southern Antarctic Peninsula over long time periods. Third, a more sophisticated elastic model, REAR, is used to correct for the effects of load changes (see section 2.3 for details).

We briefly summarize the main characteristics of the BHM, noting that further details can be found in *Zammit‐Mangion et al.* [2014, 2015a, 2015b]. The BHM adopts a two‐stage approach: (i) spatiotemporal modeling of the numerical model outputs in order to extract spectral characteristics of the processes of interest (i.e., length scales in time and space and marginal variances) and (ii) spatiotemporal modeling of the processes of interest with informative priors (based on (i)) in order to provide updated estimates. It is structured in three layers where (1) the observation layer describes the relationships between the observations and the physical processes, (2) the process layer describes the spatiotemporal evolution of the multivariate statistical model, and (3) the parameter layer contains prior information of the unknown parameters in the above two layers. In the observation layer we define the relation between the observations and the rates of elevation change attributed to each process as follows:

(1) $$\left[\begin{array}{c} {\text{y}}_{t,\text{FIRN}}\\ {\text{y}}_{t,\text{SURF}}\\ {\text{y}}_{t,\text{ICE}}\\ {\text{y}}_{t,\text{GIA}} \end{array}\right]=\text{C}(\theta )\left[\begin{array}{c} {\text{x}}_{t,\text{GRACE}}\\ {\text{x}}_{t,\text{ALT}}\\ {\text{x}}_{t,\text{GPS}} \end{array}\right]+\left[\begin{array}{c} \varepsilon_{t,\text{GRACE}}\\ \varepsilon_{t,\text{ALT}}\\ \varepsilon_{t,\text{GPS}} \end{array}\right]\;,$$

where ***x***_*t*,ALT_, ***x***_*t*,GRACE_, and ***x***_*t*,GPS_ represent vectors of equivalent elevation changes (d*h*/d*t*) recorded by altimetry, gravimetry, and GPS data, respectively. ***C***(***θ***) is an incidence matrix that maps the latent space onto the observation space, and ***θ*** is a vector of unknown parameters estimated offline [*Zammit‐Mangion et al.*, 2015a]. The latent processes: firn compaction, SMB, ice dynamics, and GIA are denoted by ***y***_*t*,FIRN_, ***y***_*t*,SURF_, ***y***_*t*,ICE_, and ***y***_*t*,GIA_, respectively. The quantities ***ε***_*t*,GRACE_, ***ε***_*t*,ALT_, and ***ε***_*t*,GPS_ are uncorrelated noise vectors. In the process layer we define spatiotemporal models for the SMB, firn compaction, ice dynamics, and GIA fields. Height changes due to the combination of these processes are modeled as Gaussian Markov random fields [*Rue and Held*, 2005] where we assume zero mean and covariance matrices (that describe the spatiotemporal variation of and interactions between the processes) which are inferred from numerical models and expert knowledge [*Zammit‐Mangion et al.*, 2015a, 2015b]. It is important to note that numerical models are only used to obtain information on the length scales, smoothness, and correlations between each process, in order to impose soft constraints on the solutions and *not* to inform the prior mean. The processes are modeled using stochastic partial differential equations which are solved using the finite element method, through triangulations of varying densities over our domain. Different resolutions are used in the meshes to solve for the different processes: finer meshes are used to resolve the short‐wavelength process (e.g., ice dynamics) as opposed to the large‐wavelength processes (e.g., GIA). The parameter layer contains the physical soft constraints used in the model which are described in detail in *Zammit‐Mangion et al.* [2015a]. The BHM used in this study has been developed as a R Software package, MVST, which is available from: https://github.com/andrewzm/MVST.

## 4 Results

### 4.1 Regional Mass Balance

**Figure 1:**
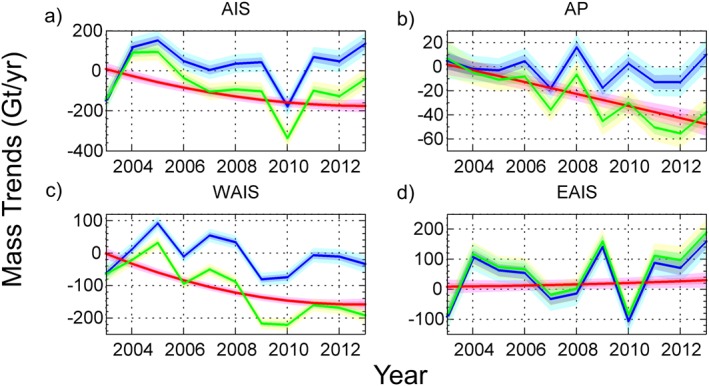
Regional mass trends for the period 2003–2013 distinguishing the SMB (blue) and ice dynamics (red) components and the total mass trend (green) for (a) Antarctic Ice Sheet, (b) Antarctic Peninsula, (c) West Antarctic Ice Sheet, and (d) East Antarctic Ice Sheet mass trends. The 1*σ* and 2*σ* confidence intervals are given by the dark and light shadings, respectively.

We estimate an average mass loss over the AIS of −84 ± 22 Gt yr^−1^ for the time period January 2003 to December 2013 (the credibility intervals here denote the 68% or 1*σ* interval). The Antarctic mass balance is characterized by strong losses from the West Antarctic Ice Sheet (WAIS) and the Antarctic Peninsula (AP) with trends of −112 ± 12 Gt yr^−1^ and −28 ± 7 Gt yr^−1^, respectively, and a significant net mass gain trend of 56 ± 18 Gt yr^−1^ for the EAIS. One of the strengths of our approach is that it allows us to assess the role of the physical processes (SMB and ice dynamics) in the overall mass balance, per drainage basin and per year. On the continental level, our results in Figure 1a show that the ice dynamics component has a strong impact on the overall mass balance of the AIS with a sustained and negative trend, but it is strongly modulated by SMB variability over the entire period, which has a standard deviation of ±105 Gt yr^−1^. The mean imbalance due to ice dynamics is −111 ± 13 Gt yr^−1^ for the 11 year period between 2003 and 2013, with a difference of −185 Gt yr^−1^ between the first and last years of our study. This component is close to balance in 2003 (8 ± 16 Gt yr^−1^), comprising −20 ± 6 Gt yr^−1^ for the ASE, compensated by thickening of Kamb Ice Stream with a magnitude of +14 ± 3 Gt yr^−1^. The SMB shows a moderate positive trend during 2003–2013, with an average of 28 ± 25 Gt yr^−1^, driven by the positive anomalies in the EAIS. However, as noted previously, has a large interannual variability [e.g., *Van den Broeke et al.*, 2011; *Lenaerts et al.*, 2012].

In the AP, the most apparent characteristic feature in Figure 1b is the development of a strong negative trend in the ice dynamics component which departs from near balance to substantial mass losses alongside no significant trend in SMB. The behavior of the WAIS is driven by the ice dynamics component exhibiting a pronounced negative trend through the entire surveyed period, although our results suggest a subtle decrease in the negative gradient in the last 4 years (Figure 1c). East Antarctica, with a net positive mass trend, is characterized by large surface mass balance variability. In contrast to the other two regions, SMB anomalies, with an average trend of 39 ± 20 Gt yr^−1^ for 2003–2013, dominate the mass balance of EAIS while the ice dynamic annual anomalies are an order of magnitude smaller in amplitude (Figure 1d), and a factor of 2 smaller in their average trend (18 ± 20 Gt yr^−1^). Our results clearly capture two strong positive SMB events in 2009 and 2011 that have been reported elsewhere [*Boening et al.*, 2012; *Lenaerts et al.*, 2013] but also an equally large negative anomaly in 2010 that has not been identified in previous studies, and which is discussed below.

**Figure 2:**
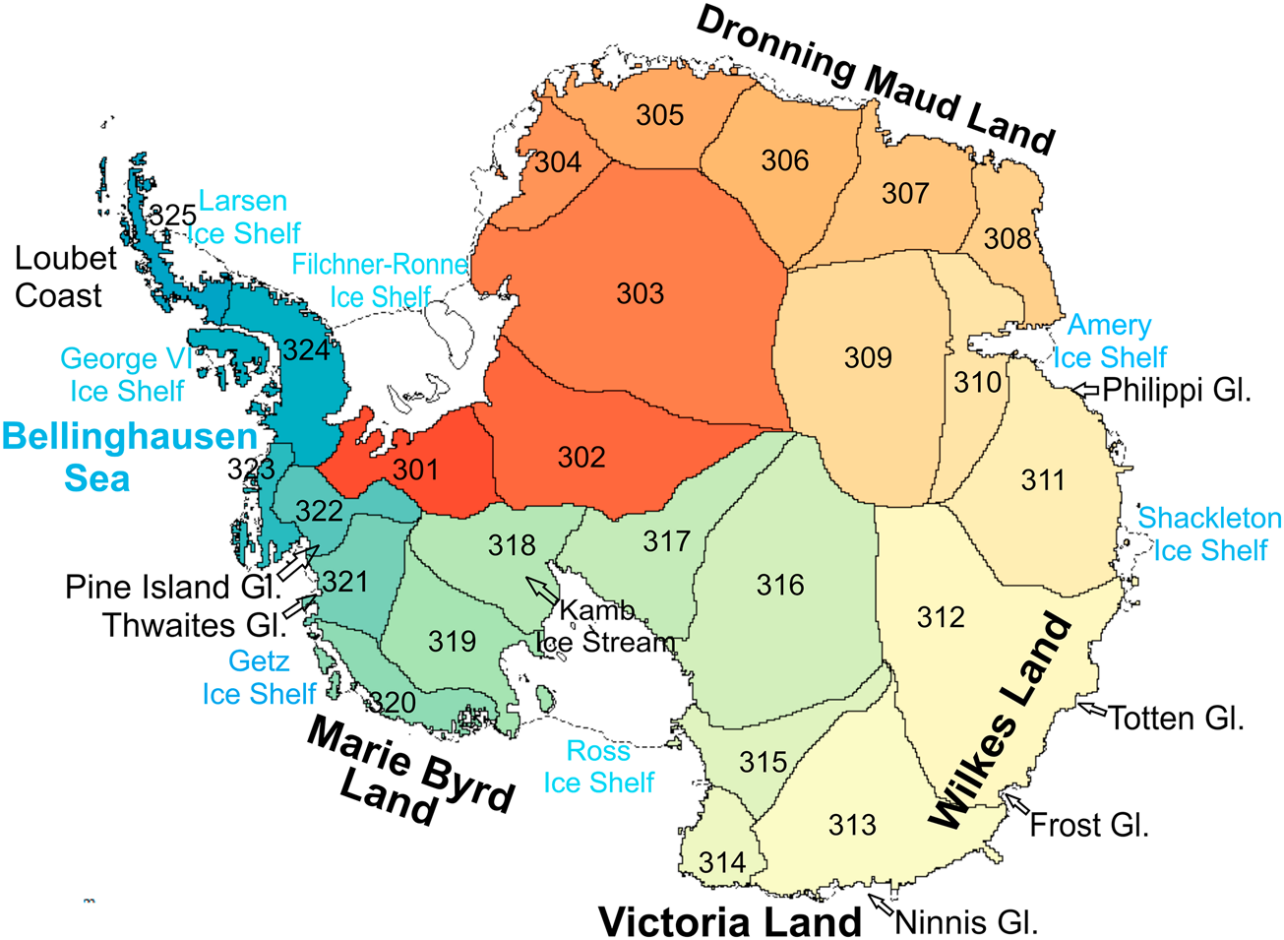
Basin locations following definitions and numbering from *Sasgen et al.* [2013], together with some specific glaciers and other landmarks mentioned in this manuscript.

**Figure 3:**
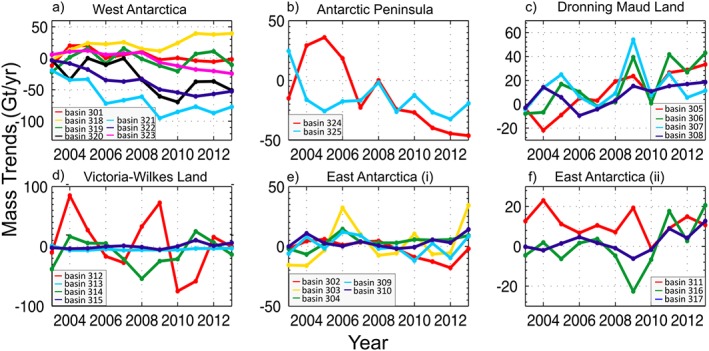
Annual mass trends on a basin level, in Gt yr^−1^. (a) Basins in the West Antarctic Ice Sheet, (b) Basins in the Antarctic Peninsula, (c) Basins in Dronning Maud Land (East Antarctica), (d) Basins in Victoria‐Wilkes Land, (e) Basins located in the upper half of East Antarctica, not included before, and (f) other basins in the lower half of East Antarctica not included before. Basin definitions and numbers corresponding to [*Sasgen et al.*, 2013].

**Figure 4:**
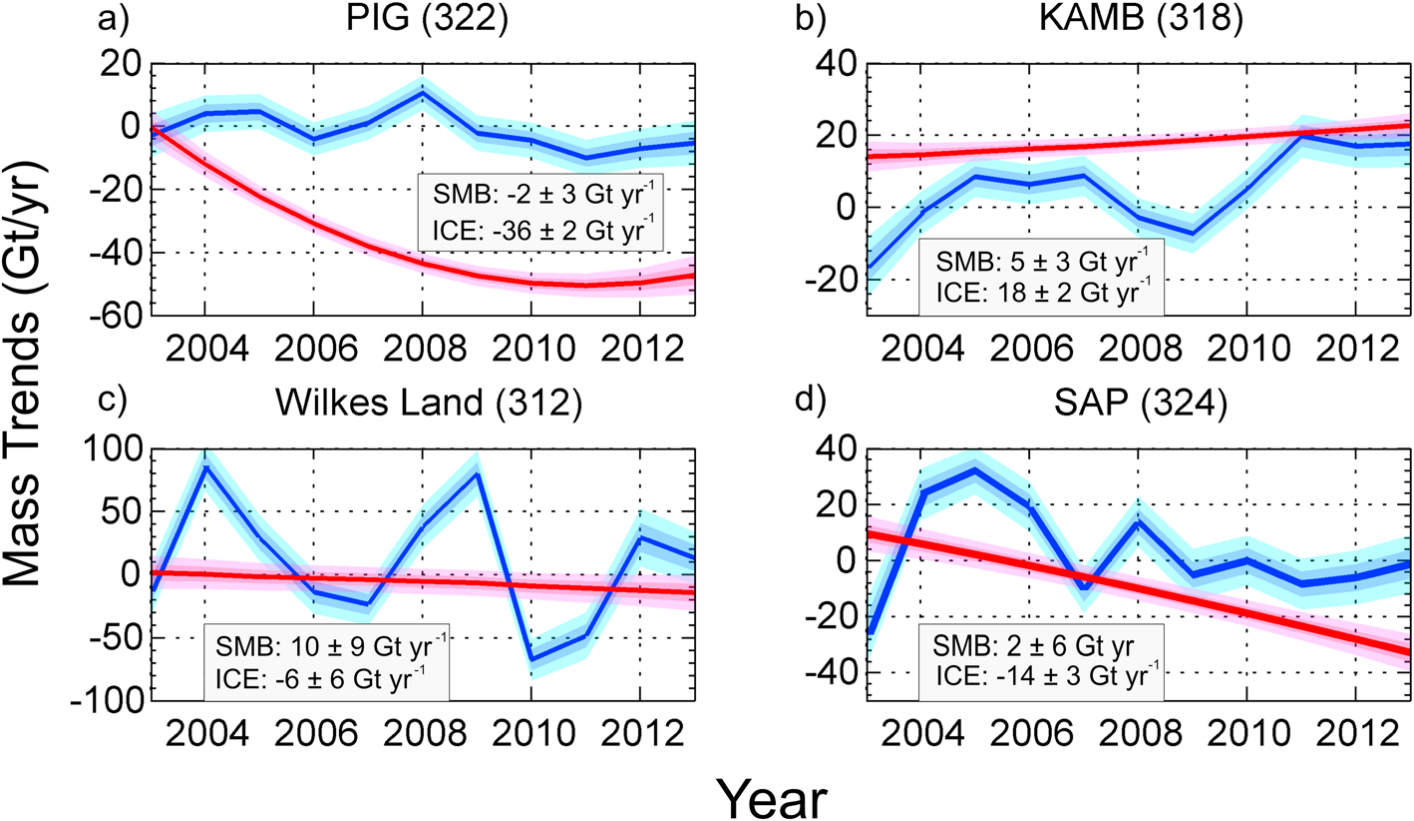
SMB (blue) and ice dynamics (red) components for (a) Pine Island Glacier (PIG, basin 322), (b) Kamb Ice Stream (KAMB, basin 318) , (c) Wilkes Land (basin 312), and (d) the Southern Antarctic Peninsula (SAP, basin 324) mass trends. The 1*σ* and 2*σ* credibility intervals are given by the dark and light shadings, respectively.

Figure 2 shows the location and definitions of the individual drainage basins of the AIS, following *Sasgen et al.* [2013], for which the estimated mass balance anomalies for different periods are summarized in Table 2. Time series of mean mass balance trends at a basin scale are shown in Figure 3 for the period 2003–2013 and Figure 4 shows the separated components of the mass balance for some particular basins. The ASE sector, in West Antarctica [basins 321 and 322, according to the *Sasgen et al.*, 2013 definitions] has experienced the strongest increase in mass loss during the surveyed period, from −56 ± 6 Gt yr^−1^ during 2003–2006 to −138 ± 6 Gt yr^−1^ during 2010–2013, bringing the average mass loss over 2003–2013 to −102 ± 6 Gt yr^−1^ (see Table 2 and light and dark blue lines in Figure 3a). Mass loss in this region is primarily due to ice dynamic processes, as shown in figure Figure 4a for Pine Island Glacier (PIG). An interesting feature seen in our results is the decrease in the ice discharge rate of PIG after 2009, following the grounding line speed stabilization during 2009–2013 [*Mouginot et al.*, 2014]. Although this sector is still losing mass, the rate appears to have modestly decelerated or plateaued in the last 2 years. The Kamb Ice Stream (318), located on the Siple Coast, is the only basin found in WAIS that exhibits a positive mass balance, 23 ± 3 Gt yr^−1^ over the period 2003–2013 (Figure 3a, yellow line). We find the ice dynamic component to be the main driver of the observed positive elevation change as reflected in Figure 4b.

**Table 2:** Average mass trends per basin for different time periods, in Gt/yr

	2003–2006		2007–2009		2010–2013		2003–2013	
Basins	Mean	SD	Mean	SD	Mean	SD	Mean	SD
301	7.2	2.9	4.5	2.6	−2.6	2.8	2.9	2.8
302	2.3	4.3	1.9	3.6	−10.5	3.6	−2.5	3.9
303	−0.8	6.4	−1.1	6.1	8.2	6.7	2.4	6.4
304	2.0	2.4	3.2	2.2	6.4	2.4	3.9	2.3
305	−7.4	3.5	15.2	3.3	23.9	3.7	10.1	3.5
306	3.1	5.0	13.1	4.9	28.0	5.2	14.9	5.1
307	10.0	4.8	20.2	4.6	12.4	4.8	13.7	4.8
308	1.9	3.6	4.7	3.4	15.3	3.6	7.5	3.6
309	3.0	5.0	2.1	4.8	‐2.7	5.2	0.7	5.0
310	3.3	3.3	0.8	3.1	5.4	3.2	3.4	3.2
311	13.4	4.9	12.3	4.6	8.4	5.3	11.3	5.0
312	21.2	7.7	26.1	7.4	−29.7	8.3	4.0	7.8
313	−3.0	5.8	−33.6	5.7	−0.9	6.3	−10.6	6.0
314	−4.3	2.0	−6.4	1.8	−4.2	2.3	−4.9	2.1
315	−2.6	2.1	−2.2	2.0	4.1	2.3	0.1	2.1
316	−1.9	4.2	−7.9	4.1	8.5	4.4	0.3	4.3
317	1.0	2.9	−1.8	2.4	6.0	2.5	2.0	2.6
318	14.3	3.1	17.4	2.6	35.3	2.9	22.8	2.9
319	−3.1	4.5	0.8	4.1	−3.3	4.4	−2.1	4.4
320	−11.7	4.9	−31.6	4.4	−48.6	4.6	−30.6	4.7
321	−39.9	4.6	−74.4	4.2	−81.7	4.5	−64.8	4.5
322	−16.0	3.6	−39.9	3.2	−56.0	3.1	−37.4	3.5
323	8.6	3.6	3.4	2.6	−18.6	3.3	−2.7	3.5
324	17.1	5.5	−15.7	5.0	−39.4	5.3	−12.4	5.3
325	−8.8	4.7	−15.1	4.5	−22.8	5.1	−15.6	4.8
EAIS	41.2	18.1	46.6	17.1	78.5	18.7	56.3	18.1
WAIS	−40.6	10.5	−119.7	9.2	−175.3	9.9	−111.9	10.1
AP	8.3	7.2	−30.7	6.8	−62.2	7.4	−28.0	7.2
AIS	8.9	22.1	−103.8	20.6	−159.2	22.4	−83.6	21.9

In East Antarctica we find two distinct patterns: Dronning Maud Land and Enderby Land (basins 305, 306, 307, and 308) show a clear positive trend (46 ± 9 Gt yr^−1^, Figure 3c), mainly driven by a positive SMB anomaly. Although the time series is too short (given the amplitude of interannual variability) to assign too much significance to the SMB trends seen here, it is interesting to note that basins 305 and 306 display a fairly monotonic increase in SMB over the 11 year period. In contrast, Wilkes Land (basin 312) displays the greatest interannual variability and appears to be largely responsible for the negative mass balance, mentioned previously, for the EAIS in 2010 (see Figure 3d). A further assessment of this basin indicates that the negative trend seen during 2010–2013 (Table 2) is mostly due to large negative SMB anomalies in 2010 and 2011 and a smaller but sustained increase of the dynamic thinning component through the whole period (Figure 4c). This dynamic thinning is consistent with increased mass losses observed in two of the main glaciers within this basin, Totten and Frost glaciers, which are grounded below sea level [*Velicogna and Wahr*, 2013] and the thinning at the glacier fronts revealed from laser [*Pritchard et al.*, 2009] and radar altimetries [*Flament and Rémy*, 2012].

Contrasted patterns are evident along the Peninsula (Figure 3b). Glaciers in the Northern Antarctic Peninsula (NAP; basin 325) had been reported to be rapidly accelerating, after the collapse of Larsen B Ice Shelf in 2002 [*Berthier et al.*, 2012]. Our results imply an accelerating mass loss in this area from 2003–2005 followed by a relatively constant rate in the remaining years of the study [*Rott et al.*, 2014]. The Southern Antarctic Peninsula (SAP) (basin 324), however, exhibited a positive mass balance of 17 ± 5 Gt yr^−1^ during 2003–2006, evident also from studies based on ICESat data [*Pritchard et al.*, 2009]. Subsequently, dynamic thinning triggered sustained, and increasing, mass loss over the outlet glaciers in this basin (Figure 4d), resulting in negative rates from 2007 onward (−16 ± 5 Gt yr^−1^ in the 2007–2009 period and −39 ± 6 Gt yr^−1^ during the consecutive time span 2010–2013) [cf., *Wouters et al.*, 2015].

**Figure 5:**
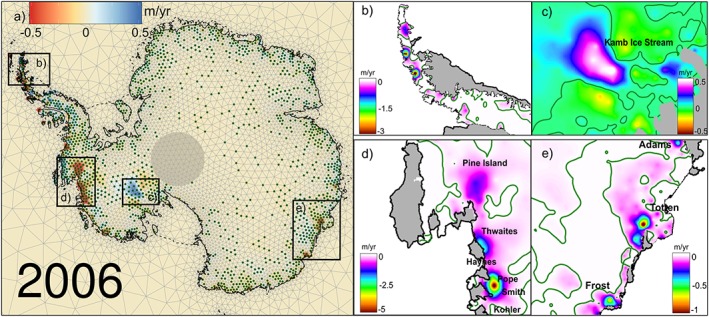
(a) Spatial pattern of surface elevation changes due to ice dynamics (stipples are shown at points where the absolute posterior mean is larger than one posterior standard deviation), (b) the Northern Antarctic Peninsula, (c) Kamb Ice Stream, (d) Amundsen Sea Embayment, and (e) Totten and Frost glaciers, where the 0 m yr^−1^ contour is displayed in green and the ice shelves are in grey.

**Figure 6:**
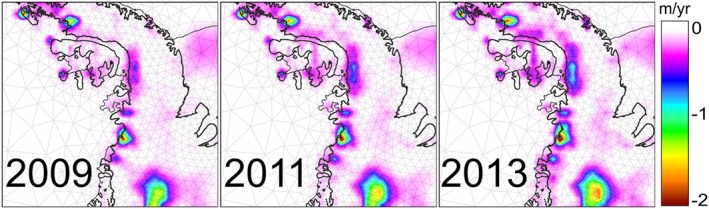
Evolution of the dynamic thinning in the Bellingshausen sector between 2009 and 2013.

We produce annual high‐resolution maps of elevation change due to ice dynamics with uncertainty estimates for the AIS. An example for 2006 is shown in Figure 5, including insets for some noteworthy regions. As expected, maximum thinning rates are found in the ASE sector in West Antarctica, which is experiencing lowering of up to 5 m yr^−1^ of ice (Figure 5, inset d) in some regions near the fronts of Smith and Pope glaciers, consistent with rapid grounding line migration noted in this sector [*Rignot et al.*, 2014] and altimetry studies [*Flament and Rémy*, 2012; *Pritchard et al.*, 2009]. Our time‐evolving solution also reflects dynamic thinning extending further along the Amundsen Sea Coast, reaching glaciers draining through the Getz Ice shelf [*Flament and Rémy*, 2012; *Pritchard et al.*, 2009]. Another well known feature seen in our solution is the positive trend over the Kamb Ice Stream (Figure 5, inset c) [*Retzlaff and Bentley*, 1993], which, here, is partly compensated by less pronounced thinning either side of this. In East Antarctica, we find several characteristic features, such as dynamic thinning on Totten Glacier, the largest outlet glacier in East Antarctica by discharge [*Rignot*, 2006; *Greenbaum et al.*, 2015] and areas surrounding Frost Glacier (Figure 5, inset e). In addition, thinning of some glaciers flowing through the Shackleton Ice Shelf became visible in 2010 [this ice shelf has been previously reported to be thinning through basal melting *Pritchard et al.*, 2012, *Flament and Rémy*, 2012], as well as in some outlet glaciers in Wilkes Land, such as Mertz and Ninnis glaciers. The Antarctic Peninsula (AP) is the most challenging region given its narrow and steep terrain, the relatively low density of altimetry measurements and the larger probability of having leakage effects [artificial low‐frequency signals resulting from the mapping of high‐frequency signals into lower frequencies *Baur et al.*, 2000] affecting GRACE measurements. Nevertheless, our statistical framework is able to resolve elevation changes that can be linked to specific glacier basins and recognizable characteristics. Substantial surface lowering is visible in the NAP along the Loubet Coast, in the former tributaries of the Larsen B Ice Shelf as well as in the glaciers flowing through the Prince Gustav Ice Shef into the Wedell Sea (Figure 5, inset b). But probably the most striking feature appears in the SAP region, along the Bellingshausen shore, where a pronounced thinning recently started to take place near the grounding line and propagated further inland, as is characteristic of dynamic thinning [*Wouters et al.*, 2015] (Figure 6). We find that the highest mass losses in this region occur at the glaciers in the English Coast that are draining into the adjacent Ferrigno Ice Stream, in the WAIS, and into southern part of George VI Ice Shelf, which was reported to have speeded up and partially broken up in 2013 [*Holt et al.*, 2013].

**Figure 7:**
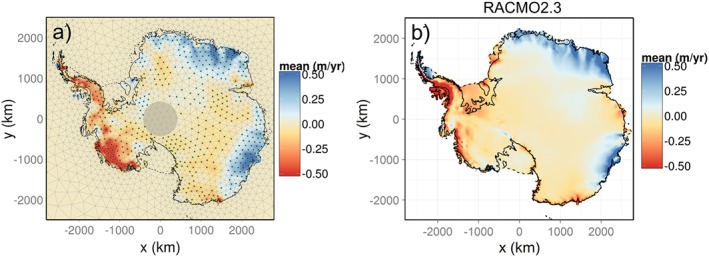
(a) Height changes caused by surface mass balance anomalies (m yr^−1^) in 2009 compared to the output from (b) RACMO2.3. Stipples are shown at points where the posterior absolute mean is larger than one posterior standard deviation. Our solutions is not displayed for the ice shelves.

**Figure 8:**
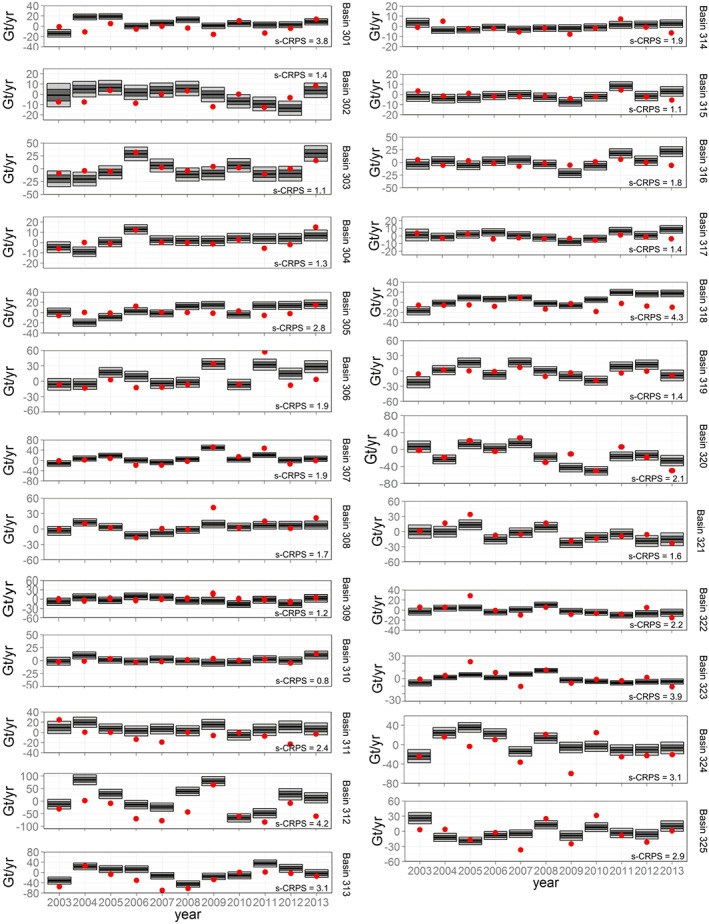
Time series of surface mass balance anomalies on a basin scale. The 1*σ* and 2*σ* credibility intervals are given by the dark and light shadings, respectively. For comparison, annual values from RACMO2.3 are shown for each basin (red dots) together with the basin‐wide standardized CRPS.

Our solution for the surface processes successfully reflects the main spatiotemporal features occurring in the AIS. To illustrate this, Figure 7 compares our SMB solution with the output from RACMO2.3 [*van Wessem et al.*, 2014] for the year 2009, showing a reasonable agreement on their spatial patterns. Over EAIS, we recover extreme snowfall events, such as the documented accumulation anomaly that took place in Dronning Maud Land in 2009 (clearly visible in Figure 7a) and in 2011 [*Boening et al.*, 2012; *Lenaerts et al.*, 2013]. The SMB estimate in this region yielded positive anomalies of 107 ± 9 Gt yr^−1^ and 73 ± 9 Gt yr^−1^ in those years, in agreement with those predicted by RACMO2.3 (basins 305–308, see Figure 8). Also in the EAIS, our results for 2009 show an exceptionally large positive anomaly in basin 312 (Wilkes Land) of 79 ± 9 Gt yr^−1^, which was also reproduced by RACMO. Negative anomalies are obtained over a large part of the ASE sector (with the exception of the coastal margin of Thwaites glacier) and in the AP. Differences in the spatial pattern of SMB anomalies can be observed in Marie Byrd Land, where our solution reveals a stronger negative trend reaching deeper inland than in the regional climate model (RCM).

Figure 8 shows the time series of SMB anomalies for the AIS at a basin scale (black bars), compared with the simulations from RACMO2.3 (red dots). To test the agreement between our estimated time series of SMB and those from RACMO, we provide the average continuous rank probability score (CRPS) coefficient for each basin [*Gneiting and Raftery*, 2007]. The CRPS is a diagnostic test that can be used to compare the posterior distribution to an observation. Here we use a standardized version of the CRPS, where the RACMO output is shifted and scaled according to the posterior mean and standard deviation of the distribution it is being compared to, and then compared to a standard normal distribution instead. This standardized CRPS is then a measure as to what extent the RACMO observations are compatible with our posterior distributions. We then take the mean of the standardized CRPSs for each basin; a large mean standardized CRPS for a basin is indicative of incompatibility between the RACMO observations and our posterior distributions for that basin, while low values imply the converse. Compare, for example, the good fit and hence low CRPS of basin 303 with the poorer fit and hence large CRPS of basins 312 and 318. Overall, we see that the temporal patterns are characterized by large interannual variability over basins within the EAIS, especially in basin 312 (Wilkes Land) where our results reveal an interannual variation up to values of 150 ± 48 Gt yr^−1^. These large amplitude changes are generally captured by the RCM, although the RACMO output is often outside of our 95% credibility intervals, as reflected by the high standardized CRPS. Basins in the WAIS and in the AP, on the other hand, present smaller interannual variabilities and generally do not show any clear trend over the study period.

Overall, we obtain good agreement with the spatiotemporal patterns predicted from the RCM at a continental scale. Mismatches at the basin scale are an expected result: RACMO is a physical interpolator that, in effect, redistributes mass entering the domain at its lateral boundaries. We would expect, therefore, a good agreement when RACMO outputs are integrated over most of the domain, while other studies have highlighted the limitations of RACMO for reproducing smaller scale spatiotemporal variability [*Medley et al.*, 2013].

### 4.2 The Amundsen Sea Embayment

**Figure 9:**
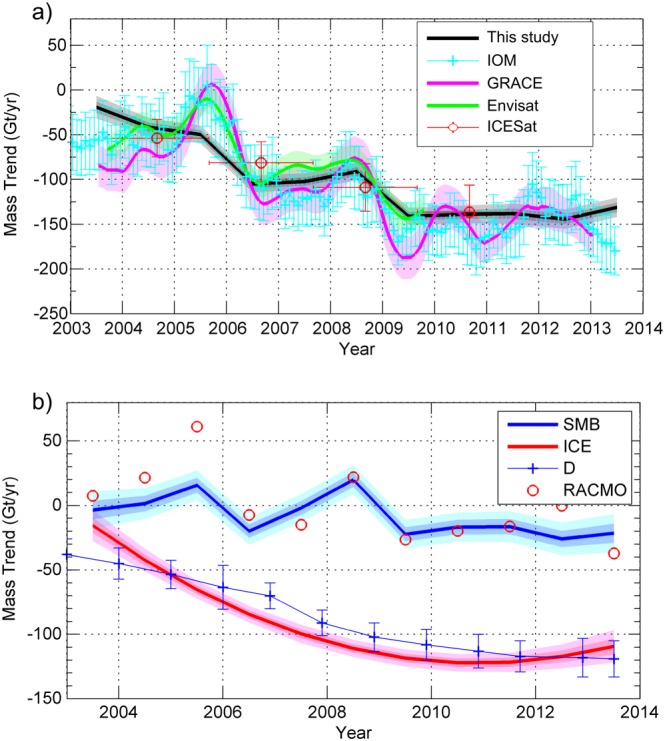
(a) Mass trends estimates for the Amundsen Sea Embayment (ASE) from our approach (black) compared with the results from four different techniques from *Sutterley et al.* [2014]: the IOM method (cyan), Gravimetry (magenta), radar altimetry from Envisat (green), and laser altimetry from ICESat (red). (b) Estimates of mass loss due to ice dynamics (red) and SMB (blue) for the ASE, compared with modeled SMB anomalies from RACMO2.3 (red dots) and ice discharge (blue line) from *Mouginot et al.* [2014]

Here we compare results from our approach with estimates from entirely independent studies and methods for an area where the signals are (i) large and (ii) well constrained. The ASE is the most suited for this purpose, having been the subject of several investigations including the acquisition of recent high‐quality field data. *Sutterley et al.* [2014] provides the most recent estimates of mass loss for the ASE sector from a combination of four different data sets (GRACE, ICESat, Envisat, and the input‐output method: IOM). Additionally, recent ice flux estimates are available for the glaciers in this sector (Pine Island, Thwaites, Haynes, Smith/Pope, and Kohler glaciers) for the period 1974–2013 using ice surface velocity and ice thickness data *Mouginot et al.* [2014]. Figure 9 displays our annual estimates of mass balance anomalies and the separated trends for ice dynamic and SMB processes for the ASE sector (comprising basins 322 and 321) compared with the estimates from the four different data sets from *Sutterley et al.* [2014]. There is, in general, a good agreement between our annual mass balance estimates and those from *Sutterley et al.* [2014], lying mainly in between the estimates from GRACE and Envisat (Figure 9a). It should be noted that some of the apparent discrepancy is due to the differences in temporal resolution between our solution (annual) and those from the other study (e.g., monthly for the IOM) (Figure 9a). The mean mass balance from the four different techniques is −84 ± 10 Gt yr^−1^ and −102 ± 10 Gt yr^−1^ for the periods 2003–2009 and 2003–2011, respectively. Our values for the same time period are slightly smaller at −81 ± 5 Gt yr^−1^ and −94 ± 6 Gt yr^−1^) but lie within the 1*σ* uncertainties. We find that the largest differences occur over the first 3 years of our study (Figure 9a), triggered by poorer availability of data during 2003 which influences the initialization of the dynamic trend (as can be inferred from Figure 9b). Nevertheless, our estimates of anomalies due to ice dynamics are compatible with the ice discharge rates estimated by *Mouginot et al.* [2014]. In particular, we obtain a decelerated rate of ice discharge in the last 2 years, an observation supported by the independent approach used in *Mouginot et al.* [2014]. On the other hand, our estimates of annual SMB anomalies correlate well with those from RACMO but less so their amplitudes. In another recent study using CryoSat‐2 data [*McMillan et al.*, 2014], the mass balance reported was −120 ± 18 Gt yr^−1^ for the time period 2010–2013. Our value for the same epoch is −138 ± 5 Gt yr^−1^, which is somewhat more negative but, again, within the 1*σ* uncertainty.

### 4.3 Glacio‐Isostaic Adjustment

**Figure 10:**
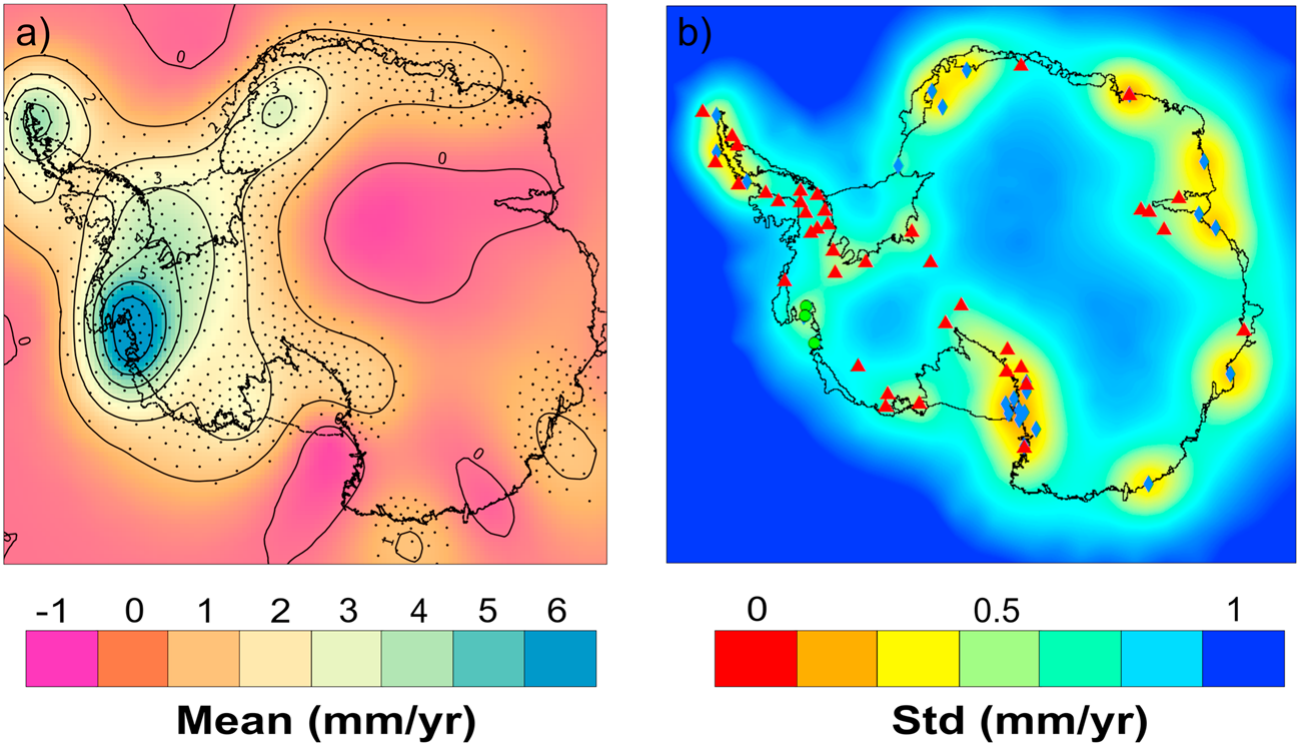
(a) Mean field of the GIA solution. Stippled points show where the absolute mean of the solution is larger than 1*σ*. (b) Posterior standard deviation of GIA Locations of the GPS stations included in this study to constrain the GIA solution is shown. Blue diamonds represent continuous GPS stations for which annual trends are derived. Red triangles show the location of non‐ideal sites (noncontinuous or campaign) stations. Green circles show the locations of the stations included in a recent study [*Groh et al.*, 2012] whose vertical velocities have been included in this study.

We obtain a temporally invariant GIA solution for the period 2003–2009. We then use this solution as a hard constraint in our framework for the full time span of this study. Figure 10 shows our GIA solution together with its uncertainty estimate and the GPS stations included in the study. Uncertainties are largest in the central parts of the ice sheet where no GPS measurements are available. Stippled points (black dots) are shown where the absolute mean of the solution is larger than 1 standard deviation. We estimate the GIA‐induced apparent mass trend to be 55 ± 8 Gt yr^−1^. The highest uplift rates are found in the ASE sector, followed by the northern part of the Peninsula and beneath the Filchner‐Ronne Ice Shelf. No significant uplift is predicted over the EAIS, with the exception of Frost and Totten glaciers in Wilkes Land, Ninnis glacier in Victoria Land, and the coastal section of Dronning Maud Land, all of which exhibit a positive GIA signal that is consistent with glaciological forward model results [*Huybrechts*, 2002; *Whitehouse et al.*, 2012].

## 5 Discussion

### 5.1 AIS Mass Balance

Our mass balance estimate for the AIS during the period January 2003 to December 2013 of −84 ± 22 Gt yr^−1^ lies in the middle range of previously published estimates. It is consistent, within the stated error bounds, with the estimate of −66 ± 44 Gt yr^−1^ obtained from a recent GRACE study [*Velicogna et al.*, 2014] for the same period. We find a discrepancy in Dronning Maud Land, however, where their study obtains larger positive anomalies (63 ± 6 Gt yr^−1^) than ours for the same region (46 ± 9 Gt yr^−1^). This discrepancy could be partially explained by the differences of the applied GIA correction in that region. In their study, the GIA signal is subtracted from GRACE using the IJ05R2 solution [*Ivins et al.*, 2013], which predicts a very small uplift over Dronning Maud Land (approximately 0.5 mm yr^−1^). In contrast, for the same region, our GIA solution estimates an average uplift of approximately 1.6 mm yr^−1^, implying that in our framework, a larger portion of the signal is due to GIA rather than by surface or ice dynamic processes. We obtain, however, a very good agreement with a global mascon GRACE solution that obtained an overall AIS trend of −81 ± 26 Gt yr^−1^ for December 2003 to December 2010 [*Luthcke et al.*, 2013] (the trend was corrected using the IJ05R2 GIA model). For the same time period we obtain −78 ± 22 Gt yr^−1^ [note that the mascon solution we use in this study is an improved and updated version of that used in *Luthcke et al.* 2013]. Good agreement is also found with a GRACE study that used a Slepian functions approach, which obtained a mass loss of −92 ± 10 Gt yr^−1^ for January 2003 to June 2014 [*Harig and Simons*, 2015]. GIA was corrected for using IJ05R2. Our estimate is less negative, although consistent within error bounds, compared with an estimate obtained from GRACE and a data‐driven GIA solution [*Sasgen et al.*, 2013] for January 2003 to September 2012 of −114 ± 23 Gt yr^−1^. On the other hand, for the same time period, our estimate is significantly more negative than the value obtained from a stochastic model that accounts for temporal correlation of regression residuals in the time series of GRACE solutions, giving a mass trend of −58 ± 16 Gt yr^−1^ [*Williams et al.*, 2014]. The overall mass trend estimated from an inversion method combining altimetry and gravimetry measurements over the epoch February 2003 to October 2009 [*Gunter et al.*, 2014] resulted in a value of −100 ± 44 Gt yr^−1^, which is considerably more negative than our results for the same period (−42 ± 24 Gt yr^−1^). A recent study using ICESat measurements estimates a net mass gain of 82 ± 25 Gt yr^−1^ between October 2003 and October 2008 [*Zwally et al.*, 2015]. This estimate strongly contrasts with previous mass balance estimates and in particular with our solution. For a similar period, we obtain a mass anomaly of −31 ± 22 Gt yr^−1^. The dominant process driving their positive mass balance is the dynamic thickening of EAIS (136 ± 50 Gt yr^−1^). Our estimate of the ice dynamics component in this region is an order of magnitude smaller that their estimates (12 ± 10 Gt yr^−1^).

One of the key benefits of our study is the partitioning of mass trends between SMB and ice dynamics. Our estimate of the dynamic imbalance rate integrated over the entire continent is nearly double (with a rate of 21 Gt yr^−2^ for the period 2003–2009) the previous estimate of *Rignot et al.* [2011], who obtained a rate of approximately 11 Gt yr^−2^ for the same time period. Interestingly, this difference does not originate from the ASE region, where our results lie at the lower end of the recent estimates of ice discharge derived from surface ice velocities [*Mouginot et al.*, 2014], (section 4.2). The discrepancy is probably due to differences in the dynamic trends of the glaciers draining into the Bellingshausen Sea and Getz Ice Shelf, where ice discharge is difficult to determine accurately due to poor knowledge of ice thickness.

Mass loss in the AP has been driven by changes on the NAP during the last two decades, after the collapse of Larsen A Ice Shelf in the 1990s. However, we find a strong negative trend in the SAP starting in 2008, eventually exceeding the losses further north [*Harig and Simons*, 2015]. Our results differ from a GRACE analysis that finds the major mass losses to be occurring in the NAP, during the period January 2003 to September 2012 [*Sasgen et al.*, 2013]. This may be a consequence of the coarse spatial resolution of GRACE. On the other hand, they agree with a recent study based on synthetic aperture radar interferometry which revealed a significant reduction in mass loss of glaciers in the NAP between 2003 and 2008, following the deceleration of glacier flow and the decrease of calving cross sections due to glacier thinning [*Rott et al.*, 2014]. Mass loss due to ice dynamics along the Bellingshausen Coast is a strong signal in our solution. This is an area where glaciers are typically grounded below sea level with a retrograde bed slope [*Wouters et al.*, 2015]. In contrast to previous analyses using CryoSat‐2 data [*McMillan et al.*, 2014], which attributed the elevation changes to negative SMB anomalies, we find dynamic thinning to be the main component driving the mass trend in this region (Figure 4a), in close agreement with the results obtained by *Wouters et al.*[2015].

As shown in Figure 8, there is good agreement between the RCM and our estimates, especially for basins in West Antarctica and in the Antarctic Peninsula, confirming the capabilities of RACMO to capture temporal patterns of surface mass balance variability. Our results show a good agreement with the observed snowfall anomalies that have been documented for Dronning Maud Land in 2009 and 2011 (basins 305, 306, 307, and 308), in the Atlantic sector of East Antarctica [*Lenaerts et al.*, 2013; *Boening et al.*, 2012]). We do not observe any unusual trends in SMB anomalies for any of the basins, with the exception of the Kamb Ice Stream catchment (Figure 8, basin 318), where the last 4 years of data reveal a positive SMB trend which has not been reproduced by RACMO.

While our estimate of the total GIA‐induced mass change (55 ± 8 Gt yr^−1^) is in good agreement with the area‐integrated GIA solution given by *Gunter et al.*[2014] from an inverse approach, and from the forward model IJ05R2 [*Ivins et al.*, 2013], our regional rates differ substantially compared with the latest forward model solutions. For Pine Island glacier, for example, we obtain a strong uplift of more than 6 mm yr^−1^ as opposed to values below 1.5 mm yr^−1^ for the GIA models IJ05R2 and AGE1. Over East Antarctica our rates are generally higher than forward models, and we obtain relatively large uplift rates over a large area of Dronning Maud Land, where subsidence is predicted from forward modeling [*Whitehouse et al.*, 2012]. Over the NAP we predict strong uplift (between 3 and 5 mm yr^−1^ over the entire region), which is considerably higher than most of the forward model rates but smaller than the uplift rates presented in *Nield et al.* [2014]. In that study the inferred spatial length scale for GIA in the AP was substantially lower (50–100 km) than the one used here (500 km), which is more in line with millennial forward GIA models such as IJ05R2 or AGE‐1b. This uplift seems to be a consequence of recent (as opposed to early Holocene) changes in ice loading, the lower viscosity of the mantle and thinner crust in the AP [*Nield et al.*, 2014]. Millennial forward GIA models do not consider recent past (centennial) changes in ice loading. As shown by *Nield et al.* [2012], these can have a large impact on GIA uplift rates, especially in regions of low viscosity and thin crust, such as the Antarctic Peninsula. The GIA solution is an improvement on earlier results obtained by *Schoen et al.* [2015] and *Zammit‐Mangion et al.* [2015b]. The main improvements are (1) a more sophisticated elastic correction has been applied to the GPS measurements in this study, (2) a change in the characteristic length scale of the GIA process, and (3) the inclusion of new GPS stations in the ASE.

### 5.2 Accuracy and Limitations

The results from the Bayesian framework are only reliable if the underlying model assumptions hold: namely, that (i) the data errors are Gaussian, uncorrelated, and correctly specified, (ii) the true processes can be treated as though they are realizations from Gaussian processes, (iii) the parameters appearing in the Gaussian process specification (namely, the length scales and the variance) are representative of the truth, (iv) the elastic model used is perfect, and (v) any mass loss or gain occurring in the pole gap does not significantly affect the final mass budget estimates. The pole gap was omitted from the final estimates due to the large uncertainties in this area.

We partially cater for (i) by estimating an observation error inflation parameter for the GRACE observations [*Zammit‐Mangion et al.*, 2015a]. In order to assess the consequences of the assumption (iii), we examine the sensitivity of our solution to a suite of prior distributions constructed from forward models (Table 3). We find that the solutions are largely insensitive to realistic parameter changes. A fixed GIA length scale (as opposed to the spatially varying one used in this study) influences the mass trends over the EAIS and the WAIS, attributing a larger mass anomaly to SMB processes. We notice a decrease in the overall uncertainties when we specify the ice model to have only linear temporal dependence. It can be shown mathematically that this response was expected. Fixing the spatial wavelength of SMB to 150 km causes a confounding between the ice and surface processes over the EAIS and little effect elsewhere. Very small changes are obtained when decreasing by one third the temporal wavelength of the firn compaction.

**Table 3:** Mass Trends for a Constant GIA Spatial Length Scale, a Linear Ice Dynamic Model in Time, a Constant SMB Spatial Length Scale, and a Smaller FIRN Temporal Correlation Parameter^a^

	Original	GIA Spatial Length	Ice Dynamics	SMB Spatial Length	Firn temporal
Region	Estimate	Scale 1700 km	Linear Model	Scale 150 km	Correlation 0.10
AIS	−83.6 ± 21.9	−81.0 ± 21.8	−87.9± 16.9	−90.1 ± 30.2	−80.5 ± 21.7
EAIS SMB	38.5 ± 19.5	32.7 ± 19.7	29.6 ± 13.9	27.2 ± 26.3	37.4 ± 21.3
EAIS ice	17.9 ± 9.8	16.7 ± 10.1	13.1 ± 10.8	17.5 ± 6.3	18.9 ± 10.9
WAIS SMB	−12.4 ± 10.3	−7.6 ± 10.2	−6.1 ± 8.7	−14.1 ± 12.2	−8.2 ± 9.9
WAIS ice	−100.9 ± 6.1	−96.4 ± 6.7	−100.5 ± 6.2	−100.3 ± 6.8	−101.5 ± 7.2
AP SMB	1.5 ± 7.1	2.2 ± 7.1	2.2 ± 6.7	0.3 ± 8.2	2.3 ± 7.6
AP ice	−29.5 ± 3.9	−28.2 ± 3.7	−26.1 ± 4.3	−28.9 ± 4.3	−29.7 ± 4.3

a All values are in Gt yr^−1^.

Assumption (ii) may be relaxed using a similar meshing approach [*Wallin and Bolin*, 2015]; however, inference with such non‐Gaussian models is at present computationally prohibitive at these scales. In order to cater for the determinacy of the elastic model (iv), we inflated the supplied variance of the GPS errors in the ASE sector, the region most susceptible to elastic effects as explained in section 2.3.

Previous works adopting this framework [e.g., *Zammit‐Mangion et al.*, 2015a] report an overall overconfidence of the results, and this overconfidence is a direct result of any or all of the assumptions made above. It is likely, however, that (i) is the strongest contributor to any incorrect uncertainty quantification since data errors remain hard to quantify (e.g., residual time‐variable penetration effects or residual ICESat campaign‐specific biases). Note that our uncertainties are even slightly smaller than those presented in *Zammit‐Mangion et al.* [2015b] as a consequence of the larger time span adopted and the lower reported uncertainties in the new data sets. This might indicate the need for more flexible time evolution models for ice dynamics; the longer the time span, the less likely a linear or quadratic function adequately fits the expected height loss due to ice dynamics. Finally, more detailed modeling effort is required for the peninsula due to the rough terrain and the relatively small size of the glaciers in that region. Our mesh is at times comparable to the magnitude of the width of the NAP which might produce underestimation of mass trends. One source of error here is the difficulty in discerning whether the loss in height attributed to a single node is due to floating ice or to grounded ice.

## 6 Conclusions

In this study we estimate the annually resolved mass balance of the AIS from a statistically based framework that isolates the different processes driving the elevation changes at any given point. The total mass loss of the AIS is −84 ± 22 Gt yr^−1^ over 2003–2013, with West Antarctica being the largest contributor with −112 ± 10 Gt yr^−1^, followed by the Antarctic Peninsula with −28 ± 7 Gt yr^−1^. These losses are partly compensated by a mass gain trend in East Antarctica of 56 ± 18 Gt yr^−1^. In addition, we provide average mass balance results for different periods (2003–2006, 2007–2009, 2010–2013, and 2003–2013) at a basin scale. Time series of SMB anomalies are calculated for each basin showing no clear trends over the surveyed period. Good agreement is found with the spatial patterns of the SMB modeled by the regional climate model, RACMO2.3, although differences arise at a basin scale. Our framework correctly separates SMB from ice dynamics in known regions of dynamic thinning (ASE) and thickening (Kamb). We have also found important changes in the dynamics in other regions, such as the beginning of a sustained negative trend in the southern part of the Antarctic Peninsula. Finally, a model independent GIA solution is obtained as a subproduct from the inversion of GPS, altimetry, and gravimetry data which could be used to constrain and validate existing and future forward models.

## Supporting information

Caption for Table S1

Table S1
