# Supplementary material for: Spatial and temporal Antarctic Ice Sheet mass trends, glacio‐isostatic adjustment, and surface processes from a joint inversion of satellite altimeter, gravity, and GPS data
Source: J Geophys Res Earth Surf. 2016 Feb 3;121(2):182–200. doi: 10.1002/2015JF003550 (PMC4845667; doi:10.1002/2015JF003550)
Supplement: Supplementary file 1 — Caption for Table S1 [file JGRF-121-182-s001.docx]

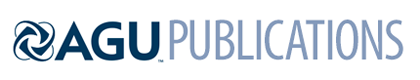


*[Journal of Geophysical Research Earth surface]*

Supporting Information for

Spatial and temporal Antarctic ice sheet mass trends,

glacio-isostatic adjustment and surface processes

from a joint inversion of satellite altimeter, gravity

and GPS data

Alba Martín-Español^1^, Andrew Zammit-Mangion^1,2^, Peter Clarke^3^, Thomas

Flament^4^, Veit Helm^5^, Matt King^6^, Scott B. Luthcke^7^, Elizabeth Petrie^8^,

Frederique Rémy^4^, Nana Schon^1^, Bert Wouters^1^, Jonathan Bamber^1^

1. School of Geographical Sciences, University of Bristol, Bristol, U.K.

2. Centre for Environmental Informatics, National Institute for Applied Statistics

Research Australia (NIASRA), University of Wollongong, Australia

3. School of Civil Engineering and Geosciences, Newcastle University, U.K.

4. LEGOS, Toulouse, France

5. Alfred Wegener Institute, Bremerhaven, Germany

6. University of Tasmania, Australia

7. NASA, Greenbelt, MD, USA

8. School of Geographical and Earth Sciences, University of Glasgow, Glasgow,

U.K.

**Contents of this file**

Caption for Tables S1

**Additional Supporting Information (Files uploaded separately)**

Table S1

**Introduction**

This file contains Supplementary Table 1 (uploaded separately) which lists all the Antarctic GPS sites used in this study, following the Digital Object Identifier implemented by UNAVCO, where available. This is a supplement of the acknowledgments section.

Table S1. List of all the GPS stations used in this study. Digital Object Identifiers given in the UNAVCO archive are stated when available.
